# Cancer Cures

**Published:** 1880-11

**Authors:** Horace R. Mooney

**Affiliations:** Taylors Creek, Geo.


ARTICLE IV.

### CANCER CURES.

COPIED BY HORACE R. MOONEY, M. D., TAYLORS CREEK, GEO.

℞ Arsenic.
Rochell Salts:
White Vitriol.
Sulphur. . . . . aa ʒi.

Mix with yolk of eggs to the consistence of butter, and put into a new earthen dish; put into a brick or stone oven; slowly bake it until it rises up higher than the top of the dish, like a well done, rich cake, let it cool and rub up fine ; mix a little of the above with the yolk of an egg and apply to the cancer.

Change every two days ; do not make the plaster quite as large as the cancer, for it will find the whole of the cancer, let it extend ever so far. Salve to use after the cancer is out :

| | | |
|---|---|---|
| Fresh butter, or lard, | . . | ℔i. |
| Beeswax, | . . . . | ℥ iv. |
| Pine turpentine, | . . . | ℥ vi. |
| Pure honey. | . . . . | ℥ ij. |
| Resin, | . . . . | ℥ iij |

Melt all together, and set off the vessel from the stove, and partly cool ; add half ounce of finely pulverized verdigris ; stir until cool.

This salve is to be used first ; afterwards the following to heal the sore :

| | | |
|---|---|---|
| Hog's lard, | . . . | ℥ iv. |
| Beeswax, | . . . | ℥ ivss. |

Melt together and stir until cool. Also for cancer, a middle receipt :

| | | |
|---|---|---|
| White Vitriol, | . . . . | 2 parts. |
| Arsenic, | . . . . | 1 part. |
| Corrosive Sublimate, | . . . . | ½ part. |

Mix the powder with simple Cerate. Mix well and apply a little of this to the cancer every day, until it is out, and heal it with the healing salve. The foregoing prescriptions enjoy great celebrity in certain parts of our State, and undoubted cures have been known to result from their use.

The practitioner will be able to decide from the nature of each case, the length of time these applications should be continued, etc.

